# Evaluation of cultural competency in a South African cluster randomised controlled trial: lessons learned for trial reporting standards

**DOI:** 10.1186/s13063-022-06767-y

**Published:** 2022-10-29

**Authors:** Nandi Louise Siegfried, Sally Hopewell, Lesley-Ann Erasmus-Claassen, Bronwyn Myers

**Affiliations:** 1 Alcohol, Tobacco and Other Drug Research Unit, South African Medical Research Council, Francie Van Zijl Drive, Parow, Cape Town, 7505 South Africa; 2 Department of Psychiatry and Mental Health, University of Cape Town, Cape Town, South Africa; 3 Centre for Statistics in Medicine, Department of Orthopaedics, Rheumatology and Musculoskeletal Sciences, Oxford Clinical Trials Research Unit, University of Oxford, Nuffield, Oxford UK; 4 Botnar Research Centre, Windmill Road, Oxford, UK; 5 Curtin enAble Institute, Faculty of Health Sciences, Curtin University, Perth, Australia; 6 Department of Psychiatry and Mental Health, University of Cape Town, Cape Town, South Africa

**Keywords:** Cultural competency, Diversity, Reporting guidelines, Randomised controlled trial, Gibbs Framework, GRIPP-2(SF) checklist

## Abstract

**Background:**

Failure to consider relevant cultural, ethnic and diversity parameters (and the intersection between these parameters) during trial protocol development and trial conduct may negatively impact recruitment, intervention development and delivery, and participant adherence and retention, potentially reducing overall internal validity. This case study aimed to evaluate the utility and comparability between the 9-item Gibbs Framework to measure cultural competency and the GRIPP-2(Short Form (SF)) 5-point checklist to assess patient and public involvement in the context of a complex clinical trial conducted in an African setting.

**Methods:**

We identified and collated all relevant publications, source and procedural data related to the trial and integrated the documents into a dynamic trial timeline. Two independent investigators applied and scored the Gibbs Framework and the GRIPP-2(SF) checklist to the four publications arising from the trial, noting functionality and comparability between tools. Where cultural competency was not met, a third investigator screened all procedural and source data and identified if cultural competency had been achieved but not reported in the publications, or if the trial had not met appropriate cultural competency based on the documentation.

**Results:**

Application of the Gibbs Framework found that the trial scored ‘2’ for seven of the nine Gibbs items, indicating full cultural competency for those questions. The Framework indicated that the trial research question was not driven by the articulated needs of patients, and neither were patients, caregivers and clinical providers involved in the development of the intervention. Comparability with the GRIPP-2(SF) checklist showed that the Gibbs performed better on evaluation of partnerships with the community, identification of culturally competent data sources and target populations, and appointment of trial staff in an inclusive manner.

**Conclusions:**

Comprehensive evaluation of the trial’s cultural competency required scrutiny of both published manuscripts and source and procedural data, suggesting that there is a gap in current trial reporting standards with respect to cultural competence.

**Trial registration:**

PACTR201610001825403. Registered on 17 October 2016.

**Supplementary Information:**

The online version contains supplementary material available at 10.1186/s13063-022-06767-y.

## Background

Cultural competence is a broad term which recognises the responsiveness of the health system to diversity of language, ethnicity and social groups [1–3]. The concept has been integrated into patient care, research and medical education in high-income countries where vulnerable and/or minority populations are relatively under-served by the health system compared to the general population [4].

To our knowledge, the term has not been widely used in the context of low- and middle-income countries (LMIC) where vulnerable, impoverished and indigent populations are likely to represent the majority of the population but may nevertheless be poorly served by the health system.

Cultural competence in the context of trial design and conduct refers to consideration of the cultural and linguistic diversity of the populations targeted for inclusion into a trial [5]. Failure to report these parameters may hamper successful implementation of effective interventions following the trial due to limitations in judging external validity (generalisability). In addition, internal validity may be affected as cultural, ethnic and diversity parameters (and the intersection between these parameters) can impact participant adherence and retention (attrition bias) and outcome measurement (detection bias). In order to optimise participant recruitment and retention in trials, identification and engagement of the target population in a trial is recognised as a key concept in cultural competency.

A checklist to guide the cultural competency of a trial may be useful to investigators during protocol development and trial reporting. Current CONSORT guidance does not include reporting related to cultural competence [6]. The 2007 Gibbs framework comprises nine criteria to evaluate the degree to which a trial meets cultural competency commencing at the community engagement stage through to analysis and dissemination [5]. The GRIPP-2(SF) is an abbreviated five-item checklist targeted to clinical trial reports [7].

This case study aims to evaluate the utility and comparability between the Gibbs and GRIPP-2(SF) tools in the context of a complex clinical trial conducted in an African setting. We retrospectively applied the 2007 Gibbs framework and the GRIPP-2(SF) checklist to source and procedural data arising from Project Mind, a three-arm cluster randomised controlled trial of 1340 patients conducted in 24 primary care clinics in both urban and rural settings in the Western Cape Province of South Africa. Project Mind compared two different systems approaches to integrating mental health and alcohol use counselling into chronic disease care with treatment as usual [8]. The target population in Project Mind was people receiving treatment for HIV or diabetes from public health services, representing the poorest, least educated, and most vulnerable populations in South Africa. Differences in education and income are likely to impact on conceptualisations of mental health and stigma, as well as views on acceptability of alcohol use. Project MIND thus presented a context in which a high degree of linguistic and cultural diversity both within sampled trial populations and between trial participants and trial investigators was implicit.

## Method

We established a Trial Cultural Competency (CC) Working Group comprising the Project MIND principal investigator (BM), the Project MIND Quality Assurance Officer (LEC), and the CC project lead who had served on the Project Mind Trial Steering Committee (NS) responsible for trial oversight and good governance. In addition, an experienced trialist and member of the CONSORT advisory group external to Project Mind (SH) was invited to join the Working Group to act as an arbiter and conduct duplicate data extraction. The study was funded by the Trial Research Methods Partnership at Liverpool University and conducted as a study within a trial [9].

To evaluate the utility and comparability between the Gibbs and GRIPP-2(SF) tools applied to Project Mind, we first identified and collated all relevant source and procedural data related to the trial. Source data included de-identified interview transcripts with facility-based staff and with patients, and procedural data such as:

- The Trial Protocol (ID: PACTR201610001825403) [10]
- The original grant application to funders
- Minutes of all trial-related meetings throughout the planning, preparation and conduct of the trial including (1) health departmental planning meetings, (2) facility-based (clinic) stakeholder meetings and (3) stakeholder advisory group meetings
- Training Manuals for the formative, pilot, and implementation phases

This documentation did not contain any patient-identifying information or trial outcome data. We also captured all publications arising from the trial, of which there were four at the time [8, 11–13]. Documentation was stored on a secure shared electronic folder in Microsoft OneDrive only available to the investigators and categorised according to the relevant phase of the trial: (1) formative, (2) pilot, (3) conduct and (4) implementation.

**Fig. 1:**
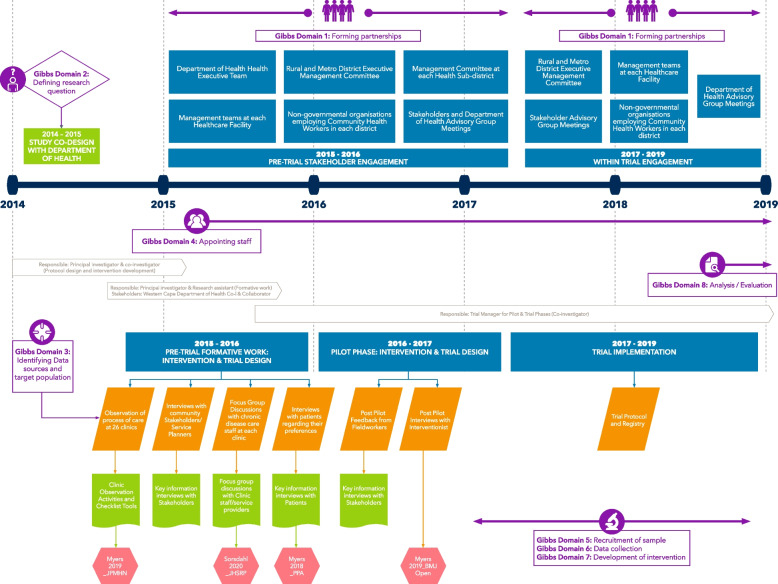
Interactive trial timeline including intercepts with Gibbs Framework Domains

An interactive trial process diagram reflecting the timeline of the trial separated by the four phases (formative, pilot, conduct, implementation) was prepared in Microsoft Visio with the responsible trial investigator identified for each phase. The source and procedural data folders were then linked electronically to the appropriate trial phase in the diagram creating an integrated and interactive data-rich timeline environment to facilitate access to the documentation required for application of the tools. See Fig. 1.

### Application of the Gibbs Framework to Project MIND

We piloted the Gibbs Framework during the South African Medical Research Council's Alcohol, Tobacco and Other Drug Research Unit journal club in which ten research staff, several of whom had worked on Project MIND, applied the Gibbs Framework to a publication reporting on data collected during the formative (early planning) phase of Project MIND [11]. Following their feedback, we added an additional clarification to the Framework guidance to permit broader interpretation of the terms ‘peer support’ and ‘community stakeholder’ to include patients, community workers and/or healthcare providers. This was considered necessary in the context of a facility-based cluster trial where the model of care to be tested required the support and acceptance of both patients and clinic staff.

We applied the tools using a two-step process, first applying both tools to the published manuscripts, and then applying the Gibbs Framework to the source and procedural data. During Step 1, two researchers (NS and SH) independently applied the Gibbs Framework and the GRIPP-2(SF) checklist to four completed published articles reporting on (1) the feasibility of conducting the trial [12], (2) a qualitative study of patient preferences for mental health counselling [13], (3) an observational evaluation of facility readiness [12] and (4) focus group and interview data with facility staff regarding optimal selection of models of integration [11]. The articles were selected for initial evaluation as the intention of both tools is application to published manuscripts. The GRIPP-2(SF) was selected over the GRIPP-2 (Long Form) as it is intended to appraise the degree of patient and public involvement in studies where patient and public involvement is a secondary or tertiary focus (such as in a clinical trial), whereas the GRIPP-2(LF) is applicable to studies in which patient and public involvement is the primary focus [7]. A spreadsheet was created in Microsoft Excel and individual judgments were recorded for each Gibbs Framework question and each GRIPP-2(SF) Checklist item.

For the Gibbs Framework, questions can be awarded as follows:

1. Culturally blind which describes methodological approaches underpinned by the belief that neither colour nor culture influence behaviour and that all people are the same
2. Culturally pre-competent describing approaches recognising that the dominant race or culture of a country is not universally applicable but failing to fully attend to cultural differences
3. Culturally competent describing approaches recognising the cultural diversity of the intended population
4. NM If there is no reporting or no information, then one should record the score as ‘Not Mentioned’ (NM)

For the GRIPP-2(SF), we recorded reporting of the item as Yes, No, or Unclear. For both instruments, we extracted supportive text from the articles and recorded these to support the judgement decisions. Agreements and discrepancies between the two coders were identified and these were then discussed before a final consensus judgement was recorded.

During Step 2, a decision-making matrix was prepared to identify those questions where it was (1) not possible to make judgments due to a lack of reporting, (2) the Gibbs Cultural Competency score was rated as ‘1’, or (3) the Gibbs Cultural Competency score was rated as ‘0’ but it was not clearly or fully reported how the researchers failed to address Cultural Competency. See Additional Tables 1 a–c. For questions meeting any of these criteria, the research assistant (LE) then systematically explored the phase-specific procedural and source data to establish whether Cultural Competency was met. The search process was ordered as follows: (1) protocols, (2) meeting minutes, (3) training manuals and (4) source data including patient and staff interview transcripts. Where additional data related to Cultural Competency was identified, a supportive extract(s) and the file name of the source document were recorded and the search process ceased. The additional data was then scrutinised by the two initial coders and a final Gibbs score was assigned based on consensus. If no elements of CC were identified after scrutinising all the available secondary and source data, the question was given a zero score for CC.

**Table 1:** Results of application of Gibbs Framework to publication, source and procedural data

No	Gibbs Domain	Gibbs explanatory question	Gibbs score following publication analysis	Additional source or procedural data analysis	Gibbs final score
1	*Forming partnerships*	Did the researchers work through gatekeepers to establish peer educators (community workers who are of the same cultural background as participants matched to the culture, language, gender, age, and life stage of the research participants)?	2	Not required	2
2	*Defining research questions*	Was the research identified and initiated by the cultural group?	1	No	1
3	*Identifying data sources and target populations*	Do the researchers recognise their own cultural framework and its influence on the research approach?	2	Not required	2
4	*Appointing staff*	Did the researchers ensure the involvement of peers, not just community gatekeepers? Are any of the following mentioned: (1) recognise cultural differences in working styles, (2) reimburse community consultants and peer educators	1	Yes	2
5	*Recruitment of sample*	Do the researchers recognise diversity within cultural groups? Do the researchers allow for the effects of acculturation over time? Are any of the following mentioned: (1) recognise potential power imbalances in working and consultation, (2) account for complexities of culture and gender, (3) recognize differences in defining language and cultural identity, (4) different understandings of research are considered to ensure informed consent, (5) offer to record verbal consent due to fear of authority	2	Not required	2
6	*Data collection*	Is the methodology responsive to cultural and migration considerations (e.g., family groups rather than individual interviews, use of professional interpreters rather than family members)	2	Not required	2
7	*Development of intervention*	Were peer educators involved in the development of the intervention? Was the intervention implemented by local community organisations?	1	Yes	1
8	*Analysis/evaluation*	Were peer educators involved in the analysis and interpretation of the data? Was there feedback from the participants to confirm the results?	2	Not required	2
9	*Reporting/disseminating findings*	Was there an opportunity for the community to discuss findings and generate solutions? Was there policy development? Were sustainable programmes developed?	2	Not required	2

Finally, the responsible trial investigators and stakeholders identified in the Trial Timeline and Process Diagram were invited to discuss the findings for the questions which were scored at the end of the process as ‘zero’ or ‘1’ and to share their reflections on Cultural Competency within the trial.

We had planned to disseminate our findings to Project MIND clinic staff and gain their views on the utility of the tool and our interpretation thereof. However, COVID-19 regulations enforced in 2021 prevented this from taking place. Due to limited internet connectivity in the health clinics, virtual dissemination was not possible.

### Comparability between the Gibbs Framework and GRIPP-2(SF)

During the application process and in monthly Working Group meetings, we identified the overlap between the instrument criteria (Gibbs and GRIPP-2(SF)) and tabulated these, recording variances and producing a graphical representation of tool comparability. We also noted challenges in coding and operationalising the guidance, and considered the utility and relevance to LMIC settings.

## Results

### Application of the Gibbs Framework and GRIPP-2(SF) to Project MIND

Results of the Gibbs and GRIPP-2(SF) evaluation of the four published manuscripts detailing the formative and pilot stages of the Project MIND trial are detailed in Additional Table 2.

**Table 2:** Utility of Gibbs Framework and suggested improvements

Utility	Improvements
Integration of CC into a systematic approach to the stages of development of a trial intervention	Create definitions for all terms used in the Framework to improve applicability e.g. expand 'peer educator' definition to include patient and staff profiles
Prompts rationale-based judgements around the levels of CC and not only binary Yes/No output	Formulate a preceding preamble to guide formulation of the target population in advance of applying the Gibbs framework
Scoring is per question permitting within-question judgments rather than an overall score for the tool (this mirrors approaches to study risk of bias assessment, e.g. the Cochrane ROB tool)	Develop an elaboration and explanation document for each question as used in CONSORT with worked examples to illustrate differences between CC levels 1 and 2
The Gibbs framework may need to be applied across multiple documents related to a single research study to fully address CC e.g. protocol, data sources, and final manuscript	Consideration to be given that a cluster trial (or other non-individually randomised trial designs) may have more than one level of target population so multiple applications of frameworks may be required targeted at the different levels of the trial population

Subsequent in-depth source and procedural data analysis to further inform the Gibbs Framework evaluation is shown in Table 1. Overall, when both the manuscript data and the source and additional procedural data were considered together, the trial was considered culturally competent scoring ‘2 s’ on all questions except questions 2 and 7.

The trial did not meet any CC criteria for question 2 viz. identification of the research question being initiated by the cultural group most likely to be affected by the trialled intervention (the patients attending the clinics).

For question 7 regarding the involvement of peers in the development of the intervention, analysis of the minutes of meetings with the provincial department of health in advance of the trial confirmed input into the intervention by departmental stakeholders only, but not of peers represented by patients, community health workers or clinical providers. The score therefore remained at ‘1’.

For question 4 which relates to involvement of peers in the appointment and reimbursement of staff, secondary data analysis revealed an additional level of cultural competency following scrutiny of the protocol submitted to the Ethics Committee (not in public domain) and the CC score was increased from ‘1’ to ‘2’. In the protocol, it was noted that community health workers were ‘already providing adherence and disease management-related counselling (and) were best placed and most receptive to being trained to deliver a brief and structured mental health intervention. These findings, together with the pre-existing body of literature on the feasibility of using community health workers to deliver mental health counselling, informed our decision to use community health workers as intervention agents’*.* In the South African context, community health workers are drawn from the patient populations that they serve and we thus considered them as ‘peers’.

### Comparability and utility of the Gibbs Framework and GRIPP-2(SF)

**Fig. 2:**
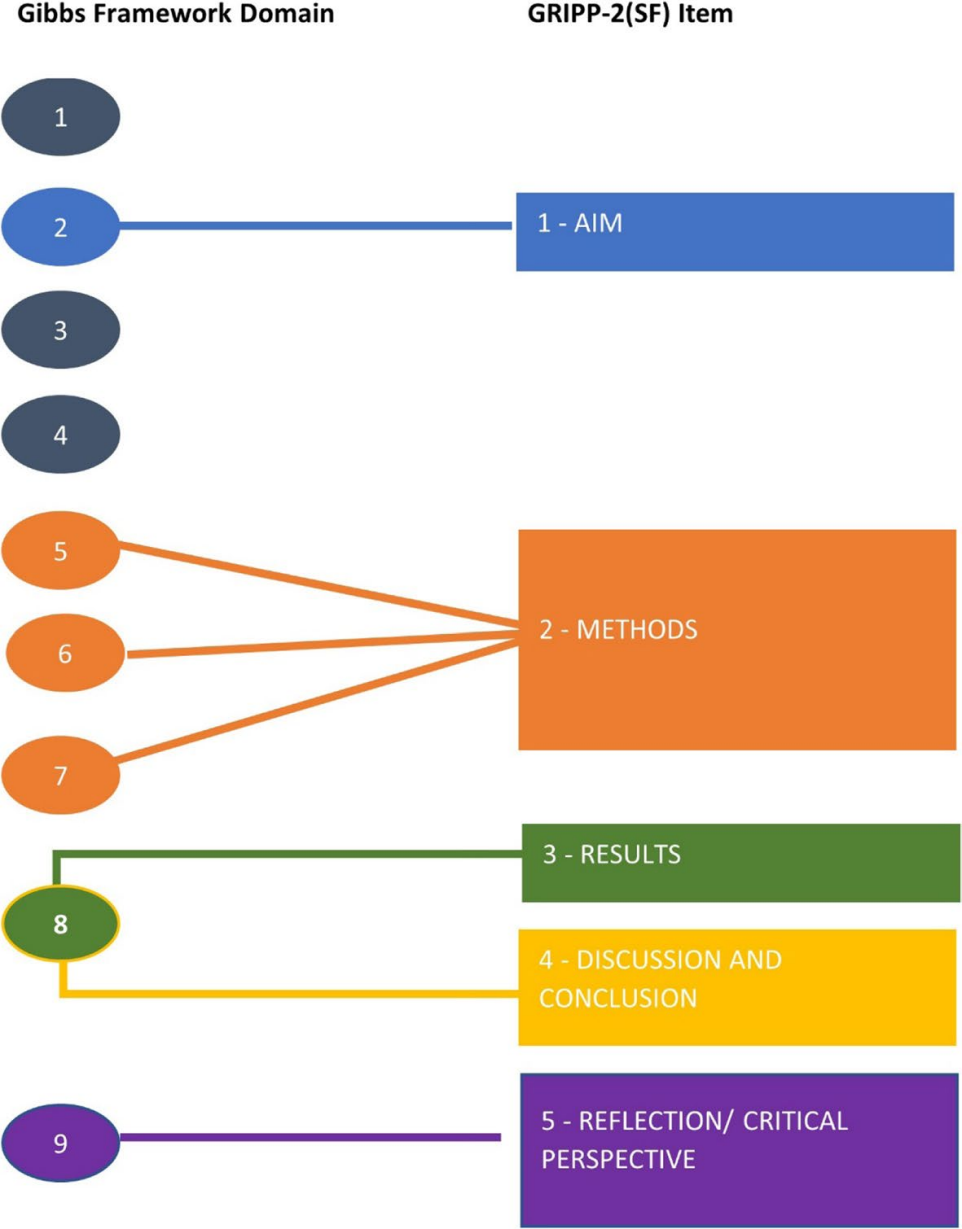
Diagrammatic representation of overlap between Gibbs Framework Domains and GRIPP-2(SF) Items

The differences and similarities between the two tools are articulated in Additional Table 3. Figure 2 illustrates the areas of overlap between the two tools. Questions 1, 3 and 4 of the Gibbs Framework are not mirrored in the GRIPP-2(SF) and cover forming partnerships, identifying data sources and target populations, and appointing staff respectively. The Working Group recorded the utility of the Gibbs Framework noting challenges and possible improvements outlined in Table 2.

## Discussion

The application of the Gibbs Framework indicated that the Project MIND trial was culturally competent in all but two of the nine Gibbs criteria. The Framework revealed that the trial research question was not driven by the articulated needs of patients, and neither were patients, caregivers and clinical providers involved in the development of the intervention. Comprehensive evaluation of the trial’s cultural competency required scrutiny of both published manuscripts and source and procedural data, suggesting that there is a gap in current trial reporting standards with respect to cultural competence.

For this study, the Gibbs Framework had distinct advantages over the GRIPP-2(SF) as it is tailored specifically to cultural competence. It includes an evaluation of partnerships forged before the start of the trial and an assessment of the awareness of the investigators’ cultural framework and its influence on their research approach. Self-reflection of a researcher’s own cultural bias is a key component of the qualitative research paradigm [14] but is rarely considered in the conduct or reporting of a clinical trial. This may be lacking in the clinical trialist’s toolbox of skills and may be especially important for trialists working in countries and cultural settings different from their own. Failure to consider the lens of the trialist and how it may differ to those of the trial participants and indeed to that of the trial clinical staff may impact successful recruitment, participation, and ultimately the robustness of findings if attrition is high.

We can foresee the criteria of the Gibbs framework being used to guide all stages of framing the trial research question, protocol development and final analysis. Our view is supported by a recent systematic review in which modified Gibbs criteria were applied to 80 HIV adherence trials mainly conducted in the USA [1]. The authors observed a lack of culturally competent trial methods but noted that inadequate reporting hampered their ability to be conclusive. Identification of the key components of the Gibbs to incorporate into the current CONSORT Statement [15] and potentially the SPIRIT Statement [16] which guide reporting standards for trial conduct and protocol development respectively, will clearly require further interrogation, development, and collaboration among trialists. Consideration will need to be given to the implications of any such changes to the stability of CONSORT Extension Statements such as that of CONSORT-SPI which pertains to social and psychological interventions [17]. We believe the Gibbs Framework is a reasonable starting point for these discussions.

In the USA, cultural competence, as a construct to incorporate appropriate responsiveness to differences in ethnicity, language and other cultural markers, has been criticised for failing to recognise the extent of systemic racism inherent in healthcare [4]. In many of these settings, vulnerable populations represent migrants, immigrants and other minority populations. This is in direct contrast to a country like South Africa where the vast majority of the general population are impoverished and experience vulnerability. In settings where clinical researchers may have low exposure to, and awareness of, linguistic and other diversity indicators specifically in minority populations, a tool like the Gibbs Framework may be an important step in furthering responsiveness in trial conduct.

Following the fall of apartheid in South Africa and the emergence of the HIV epidemic, clinical research developed a strong social justice and advocacy approach [18]. Community preparedness studies are conducted prior to clinical and public health trials, and almost all HIV trials conducted in the public sector include regular input from Community Advisory Boards (CAB), ensuring active participation by those likely to be most affected by the interventions under trial [19, 20]. As a result, the terminology of cultural competency (and its inherent ‘othering’) may not be the most appropriate for our setting where diversity and the importance of ensuring inclusivity and equity are encouraged in research. Nevertheless, application of the Gibbs Framework to the Project Mind trial did elicit important deficits with the lack of participation by the patient population in driving the research question being the most critical. This was also observed in the HIV trials included in the systematic review referred to earlier [1] and is not addressed by the scope of a CAB or by community preparedness.

In applying the Gibbs Framework, we identified several challenges including the lack of clear definitions for many of the terms used in the tool and recommend that an updated version of the tool consider the inclusion of a user glossary. This should be informed by scrutiny of the terminology used across a wide range of clinical and public health settings and regions to reflect and achieve greater global reach. In addition, real trial examples which illustrate the differentiation between thresholds of cultural blindness through to cultural competence will greatly aid in ensuring consistent scoring between researchers.

Our approach made use of both internal and independent evaluators working in partnership, as described by Conley-Tyler [21], to gain the benefits of both approaches. Given the need to access unpublished data to fully assess cultural competency and to understand the trial context, it was advantageous to include trial investigators in the project team. We aimed to address any real or perceived subjectivity by having all scoring conducted by two independent authors, one of whom was not involved in the Project MIND trial in any capacity and was considered impartial. Final decisions were made collectively to ensure the robustness of the results. The use of shared software and the creation of an interactive trial timeline linked to manuscripts, source data and procedural data greatly enhanced our ability to conceptualise all the stages of the Project MIND trial where cultural competence was important to consider. Generalisability of our results is limited given that we applied the Framework to a single trial. However, as a clinic-based cluster trial incorporating both clinical and behavioural outcomes, we believe Project MIND provided a sufficiently complex platform to thoroughly evaluate the Gibbs tool.

Our study included a global team with research expertise in reviewing and conducting clinical trials in both low and high-income settings. As a team, we share limited anthropological knowledge and experience and recognise that expanding this field of research will require a more diverse inter-disciplinary approach. During our discussions, we consciously created an environment where questions regarding diversity within the clinic setting were candidly explored. We acknowledge that our lived experiences of navigating the healthcare research environment are vastly different to those of the participants in Project Mind and note that future teams should be inclusive of patient and provider representation in equal measures to researchers.

## Conclusion

Our approach operationalised secondary data analytical methods for the application of the Gibbs Framework to a LMIC trial which was judged to be culturally competent in seven of nine domains. The process and results will be important to repeat and replicate to inform revision of the Framework to improve its functionality and usability. Further evaluation may take the form of retrospective application to completed trials, such as in our study, or prospective application in planned and ongoing trials while monitoring the utility of its use. Comprehensive evaluation of the trial’s cultural competency required scrutiny of both published manuscripts and source and procedural data, suggesting that there may be a gap in current trial reporting standards with respect to cultural competence.

## Supplementary Information

**Additional file 1: Supplementary Table S1a.** Guide to Publication reporting rating. **Supplementary Table S1b.** Source and procedural data to be searched and scrutinised for further data. **Supplementary Table S1c.** Matrix of Gibbs score and publication reporting to determine data scrutiny.

**Additional file 2: Supplementary Table S2.** Results of application of Gibbs Framework and GRIPP-2(SF) to publications from Project MIND.

**Additional file 3: Supplementary Table S3.** Comparison between the Gibbs Framework and the GRIPP-2(SF).

## Data Availability

The dataset supporting the conclusions of this article is included within the article (and its additional files).
