# Supplementary material for: Evaluation of cultural competency in a South African cluster randomised controlled trial: lessons learned for trial reporting standards
Source: Trials. 2022 Oct 29;23:918. doi: 10.1186/s13063-022-06767-y (PMC9617747; doi:10.1186/s13063-022-06767-y)
Supplement: Supplementary file 1 — Additional file 1: Supplementary Table S1a. Guide to Publication reporting rating. Supplementary Table S1b. Source and procedural data to be searched and scrutinised for further data. Supplementary Table S1c. Matrix of Gibbs score and publication reporting to determine data scrutiny. [file 13063_2022_6767_MOESM1_ESM.docx]

**Supplementary Table S1a – Guide to Publication reporting rating**

| **Publication Rating** | **Guide** | **Notes** |
| --- | --- | --- |
| **YES** | The answer is reported in the document permitting a score of 0, 1 or 2 | Note that a question can be scored a ZERO (no CC), but the answer is reported in the document (e.g. the peer educators were clearly not initiating the study and the document reports or refers to this e.g. the trial was initiated by the researchers) |
| **NO** | There is no report for or against the answer | This is then linked in the Gibb's tool as 'NO MENTION' |
| **UNCLEAR** | There is some mention in the document regarding the question, but it is not clear how the researchers addressed CC or it is insufficient to judge |  |

**Supplementary Table S1b - Source and procedural data to be searched and scrutinised for further data:**

| **Gibbs** | **Publication reporting** |
| --- | --- |
| 0 | Unclear |
| 1 | Yes / Unclear |
| No Mention (NM) | No |

**Supplementary Table S1c – Matrix of Gibbs score and publication reporting to determine data scrutiny**

| **Gibbs** | **Publication reporting** | | |
| --- | --- | --- | --- |
| 0 | Yes | _ | Unclear |
| 1 | Yes | _ | Unclear |
| 2 | Yes | _ | _ |
| NM | _ | No | _ |
| **Notes:**   1. All red blocks will require source and procedural data scrutiny | | | |
| 1. It is impossible to select ‘No’ for publication reporting if you rate a Gibbs as 0, 1 or 2 | | | |
