# Supplementary material for: Evaluation of cultural competency in a South African cluster randomised controlled trial: lessons learned for trial reporting standards
Source: Trials. 2022 Oct 29;23:918. doi: 10.1186/s13063-022-06767-y (PMC9617747; doi:10.1186/s13063-022-06767-y)
Supplement: Supplementary file 2 — Additional file 2: Supplementary Table S2. Results of application of Gibbs Framework and GRIPP-2(SF) to publications from Project MIND. [file 13063_2022_6767_MOESM2_ESM.docx]

**Supplementary Table S2: Results of application of Gibbs Framework and GRIPP-2(SF) to publications from ProjectMIND**

| **Myers 2018 PPA** | | | |  | **Myers 2019 BMJ Open** | | | |
| --- | --- | --- | --- | --- | --- | --- | --- | --- |
| **Gibbs Criteria** | **Final score** | **GRIPP-2(SF) items** | **Final decision** |  | **Gibbs Criteria** | **Final score** | **GRIPP-2(SF) items** | **Final decision** |
| 1 | NM |  | |  | 1 | NM |  | |
| 2 | NM | 1 | Yes |  | 2 | 1 | 1 | Yes |
| 3 | NM |  | |  | 3 | NM |  | |
| 4 | NM |  |  |  | 4 | 1 |  |  |
| 5 | 1 | 2 | Yes |  | 5 | 1 | 2 | No |
| 6 | 2 |  |  |  | 6 | 2 |  |  |
| 7 | 1 |  |  |  | 7 | 1 |  |  |
| 8 | NM | 3 | Yes |  | 8 | 2 | 3 | No |
| 8 | NM | 4 | Yes |  | 8 | 2 | 4 | No |
| 9 | NM | 5 | Yes |  | 9 | 2 | 5 | Unclear |
|  |  |  |  |  |  |  |  |  |
| **Myers 2019 JPMHN** | | | |  | **Sorsdahl 2020 JHSRP** | | | |
| **Gibbs Criteria** | **Final score** | **GRIPP-2(SF) items** | **Final decision** |  | **Gibbs Criteria** | **Final score** | **GRIPP-2(SF) items** | **Final decision** |
| 1 | 2 |  | |  | 1 | NM |  | |
| 2 | NM | 1 | No |  | 2 | NM | 1 | No |
| 3 | 2 |  | |  | 3 | 1 |  | |
| 4 | 1 |  |  |  | 4 | NM |  |  |
| 5 | 1 | 2 | Yes |  | 5 | 2 | 2 | No |
| 6 | NM |  |  |  | 6 | 2 |  |  |
| 7 | NM |  |  |  | 7 | NM |  |  |
| 8 | 1 | 3 | No |  | 8 | NM | 3 | No |
| 8 | 1 | 4 | Yes |  | 8 | NM | 4 | No |
| 9 | 1 | 5 | Yes |  | 9 | 2 | 5 | No |
