# Supplementary material for: Evaluation of cultural competency in a South African cluster randomised controlled trial: lessons learned for trial reporting standards
Source: Trials. 2022 Oct 29;23:918. doi: 10.1186/s13063-022-06767-y (PMC9617747; doi:10.1186/s13063-022-06767-y)
Supplement: Supplementary file 3 — Additional file 3: Supplementary Table S3. Comparison between the Gibbs Framework and the GRIPP-2(SF). [file 13063_2022_6767_MOESM3_ESM.docx]

**Supplementary Table S3: Comparison between the Gibbs Framework and the GRIPP-2(SF)**

| **GIBBS** | | | **Comparison Notes** | **GRIPP-2** | | |
| --- | --- | --- | --- | --- | --- | --- |
| **No** | **Gibbs Domain** | **Gibbs explanatory question** |  | **No** | **GRIPP-2SF Domain** | **GRIPP-2 questions** |
| 1 | *Forming partnerships* | Did the researchers work through gatekeepers to establish peer educators (community workers who are of the same cultural background as participants matched to the culture, language, gender, age, and life stage of the research participants)? | The GRIPP2-SF can be used when reporting public involvement in a study; but mainly used in a retrospective manner by most researchers "as a quality assurance step in the writing up of PPI in publications and reports". The GRIPP2_SF thus look at forming partnerships in a completely different manner than the Gibbs does, but also because the GRIPP2-SF is mainly focused on patient and public involvement (PPI) in health/clinical and social care research. Gibbs is based on principles of community-based participatory research and thus recognizes cultural influences and/or differences at a much earlier stage and its particular beneficial during the proposal writing stage where the researcher can take into consideration all cultural related elements such as whether staff and participants would be matched culturally or in terms of gender, age, language, etc. |  |  |  |
| 2 | *Defining research questions* | Was the research identified and initiated by the cultural group? | The difference in how this question is framed in both Gibbs and GRIPP2_SF is indicative of then they are applied to the text. Gibbs can be applied before and during a study whereas GRIPP-SF and GRIPP2-LF provides guidance in terms of reporting on a study. When reading about the development of GRIPP2-SF (Table 3; pg 4) it mentions that under the "Aim" section the checklist was meant to determine whether the study collaboratively involved patients as research partners at all stages. | 1 | *Aim* | Is the aim of PPI reported in the study? |
| 3 | *Identifying data sources and target populations* | Do the researchers recognize their own cultural framework and its influence on the research approach? | Definition of who the target population would be seems to be differently viewed in the Gibbs and the GRIPP2-SF |  |  |  |
| 4 | *Appointing staff* | Did the researchers ensure involvement of peers, not just community gatekeepers? Are any of the following mentioned: 1) Recognize cultural differences in working styles 2) Reimburse community consultants and peer educators | This item for Gibbs is not taken into consideration by the GRIPP2-SF or written in a clear manner |  |  |  |
| 5 | *Recruitment of sample* | Do the researchers recognize diversity within cultural groups? Do the researchers allow for effects of acculturation over time? Are any of the following mentioned: 1) Recognize potential power imbalances in working and consultation 2) Account for complexities of culture and gender 3) Recognize differences in defining language and cultural identity 4) Different understandings of research are considered to ensure informed consent 5) Offer to record verbal consent due to fear of authority | GRIPP2-LF provides more depth to this section where it looks at (a) design; (b) people involved; (c) stages of involvement; and (d) level or nature of involvement which is similar to how Gibbs questions how the researcher looked cultural considerations when recruiting participants. When reviewing the different publications linked to project MIND it was a topic of discussion to determine who would be considered as the actual participant. According to the Gibbs the participant would be considered as the healthcare providers but for the GRIPP2-SF the PPI or participant would be the actual patients who received the counselling intervention | 2 | *Methods* | Is there a clear description of the methods used for PPI in the study? |
| 6 | *Data collection* | Is the methodology responsive to cultural and migration considerations (e.g., family groups rather than individual interviews, use of professional interpreters rather than family members) | GRIPP2-SF Methods section |  |  |  |
| 7 | *Development of intervention* | Were peer educators involved in the development of the intervention? Was the intervention implemented by local community organizations? | GRIPP2-SF Methods section |  |  |  |
| 8 | *Analysis/evaluation* | Were peer educators involved in the analysis and interpretation of the data? Was there feedback from the participants to confirm the results? | Both the "Study results" and "Discussion and Conclusions" sections from the GRIPP2-SF seems to cover the "Analysis/evaluation" section of the Gibbs. | 3 | *Study results* | Outcomes: Were the results of PPI in the study, including both positive and negative outcomes, reported? |
|  |  |  |  | 4 | *Discussion and Conclusions* | Outcomes: Did the investigators comment on the extent to which PPI influenced the study overall? Did they describe positive and negative effects? |
| 9 | *Reporting/disseminating findings* | Was there an opportunity for the community to discuss findings and generate solutions? Was there policy development? Were sustainable programs developed? | The "Report/disseminating findings" section from the Gibbs and the "Reflections/critical perspective" section from the GRIPP2-SF are the only sections that are really comparable since it looks at the potential impact the research has had, how it was reported on and whether the researchers has critically reflected on the work which has been completed. | 5 | *Reflections/critical perspective* | Did the investigators comment critically on the study, reflecting on the things that went well and those that did not, so others can learn from this experience? |
